# Structural and Optical Properties of NiO/ZnS Core–Shell Nanostructures for Efficient Quantum Dot Light-Emitting Diodes

**DOI:** 10.3390/ma16145106

**Published:** 2023-07-20

**Authors:** Jungho Kim, Jiwan Kim

**Affiliations:** Department of Advanced Materials Engineering, Kyonggi University, Suwon 16227, Republic of Korea

**Keywords:** colloidal quantum dots, NiO, NiO/ZnS, quantum dot light-emitting diodes

## Abstract

Colloidal quantum dots (QDs) have emerged as promising candidates for optoelectronic devices. In particular, quantum dot light-emitting devices (QLEDs) utilizing QDs as the emission layer offer advantages in terms of simplified fabrication processes. However, the use of poly(3,4-ethylenedioxythiophene):poly(styrene-sulfonate) as a hole injection layer (HIL) in QLEDs presents limitations due to its acidic and hygroscopic nature. In this study, NiO/ZnS core–shell nanostructures as an alternative HIL were studied. The ZnS shell on NiO nanoparticles effectively suppresses the exciton quenching process and regulates charge transfer in QLEDs. The fabricated QLEDs with NiO/ZnS HIL demonstrate high luminance and current efficiency, highlighting the potential of NiO/ZnS as an inorganic material for highly stable all-inorganic QLEDs.

## 1. Introduction

Colloidal quantum dots (QDs) are semiconductor particles that exist in nanoscale colloidal form. QDs exhibit a unique property known as the quantum confinement effect, which enables the precise control of the emission wavelength. QDs are highly suitable for various optoelectronic devices owing to their excellent color purity, high internal quantum efficiency, and solution processability [1,2]. Quantum dot light-emitting devices (QLEDs) do not require a vacuum system to form the emission layer (EML), simplifying the fabrication process. Therefore, QDs are gaining considerable attention for future applications in display technology. Using cadmium (Cd)-based QDs, the external quantum efficiencies (EQE) of red, green, and blue devices exhibited 30.9, 23.9, and 19.8%, respectively [3,4,5]. However, due to the toxicity of Cd, eco-friendly QD materials are recently gaining attention for consumer display electronics. In 2019 and 2020, Jang et al. achieved an EQE of 21.4 and 20.2% with red indium phosphide- and blue zinc selenide-based QDs, respectively [6,7].

In standard QLED structures, a large hole injection barrier exists between the indium tin oxide (ITO) electrode and the hole transport layer (HTL). Poly(3,4-ethylenedioxythiophene):poly(styrene-sulfonate) (PEDOT:PSS) exhibits excellent electrical conductivity, optical transparency, and a high work function, which are tremendous advantages for effective hole injection. However, practical applications of PEDOT:PSS are limited because of its acidic and hygroscopic nature, which can corrode the ITO layer [8]. Therefore, chemically stable inorganic materials that can replace PEDOT:PSS are required to improve the efficiency and stability of QLEDs. Several studies have been performed to investigate metal oxides such as CuO, MoO_x_, and V_2_O_5_ as hole injection layers (HILs) for QLEDs [9,10,11]. However, the performance of these QLEDs did not match that of devices with PEDOT:PSS as the HIL.

Nickel oxide (NiO) is an intrinsic p-type semiconductor and has excellent hole injection and electron blocking capabilities due to its deep valence-band maximum (VBM) and wide bandgap [12,13]. Therefore, NiO-based HTLs have been used for various solar cells replacing degrading organic materials [14,15,16]. However, only a limited number of studies on electroluminescence (EL) devices with NiO have been reported [17,18]. In this study, we further applied a ZnS shell to NiO as a core–shell structure for use as a HIL in QLEDs. The ZnS shell on NiO stabilized the surface defects of the core and controlled the charge transfer in the QD. Zinc Sulfide (ZnS) shells were successfully formed on NiO nanoparticles (NPs) using a sol–gel method. Additionally, various optical and structural properties of NiO/ZnS were investigated to confirm its stability as a HIL for QLEDs. The devices using NiO/ZnS thin films as the HIL of the QLEDs exhibited peak luminance and EQE of 4068.5 cd/m^2^, 1.23%, respectively. These results demonstrate that NiO/ZnS is a promising alternative inorganic material for HIL and could facilitate the fabrication of highly stable all-inorganic QLEDs.

## 2. Materials and Methods

### 2.1. Synthesis of NiO/ZnS Core–Shell Nanostructure

**Figure 1:**
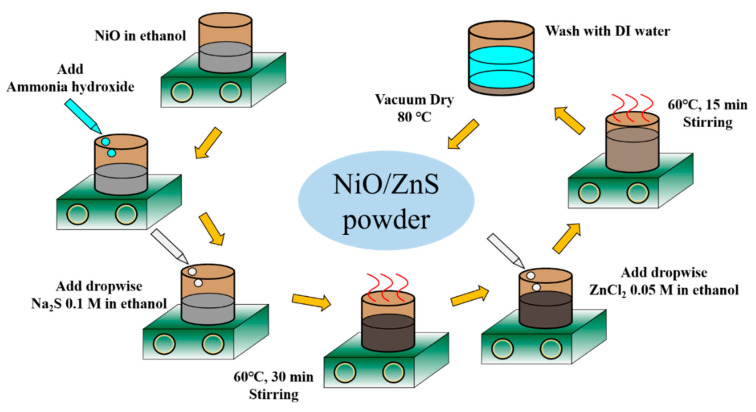
Schematic of the synthesis of NiO/ZnS nanostructures.

A schematic of the synthesis of the NiO/ZnS nanostructures is shown in Figure 1. The NiO solution was prepared by dispersing 0.3 g of NiO NPs (Sigma Aldrich, St. Louis, MO, USA) in ethanol. After the pH was adjusted to 10 using ammonium hydroxide, a solution of 0.1 M Na_2_S in ethanol was added dropwise to the NiO solution. After stirring at 60 °C for 2, 3, or 4 h, a solution of 0.05 M ZnCl_2_ in ethanol was added dropwise into the resultant mixed solution and stirred for half the previous stirring time. The product was then washed with deionized water and dried at 80 °C.

### 2.2. Synthesis of Green-Emitting QDs

Green-emitting CdZnSeS/ZnS QDs were prepared using the synthesis procedure described in the previous publication [19]. Briefly, Cd oxide and Zn oxide were placed with oleic acid (OA) in a three-neck flask and heated to 150 °C with N_2_ flowing. Then, 1-octadecene (ODE) was added and heated to 310 °C. Subsequently, a Se+S stock solution in trioctylphosphine (TOP) was swiftly injected into the mixture, and the reaction of the composition-gradient CdZnSeS core was carried out at 310 °C. For a successive ZnS shelling, Zn acetate dihydrate dissolved in OA and ODE was rapidly put to the above reactor and the reaction was held at 270 °C. Then, S dissolved in TOP was added dropwise, followed by the ZnS reaction at that temperature. The resulting green-emitting CdZnSeS/ZnS QDs were repeatedly purified via centrifugation with a solvent/non-solvent combination.

### 2.3. Fabrication of QLEDs

For device fabrication, ITO-coated glass substrates were first cleaned via ultrasonication with isopropyl alcohol and deionized water. Before the deposition of the HIL, the substrates were cleaned using UV–ozone treatment for 15 min to generate a hydrophilic surface. Subsequently, NiO/ZnS in ethanol (25 mg/mL) was spin-coated on the substrate as the HIL at 3000 rpm for 60 s, and then annealed in air at 70 °C. The coated substrates were then transferred to an N_2_-filled glove box for spin-coating the poly[(9,9-dioctylfluorenyl-2,7-diyl)-co-(4,4′-(N-(4-sec-butylphenyl)diphenylamine)] (TFB) and QD layers. The TFB layer was spin-coated at 2000 rpm for 35 s using an 8 mg/mL solution in chlorobenzene, and then baked at 150 °C for 30 min. Thereafter, a suspension of CdZnSeS/ZnS QDs in hexane (10 mg/mL) was spin-coated at 2000 rpm for 20 s. To deposit the electron transport layer and cathode, the substrates were transferred into a thermal evaporator and TPBi (40 nm), LiF (1 nm), and Al (100 nm) layers were thermally deposited sequentially under a base pressure of 5 × 10^−6^ Pa. Finally, the devices were immediately encapsulated in glass by using an ultraviolet sealant in the glove box. For comparison, a reference device was prepared using the same fabrication process with the NiO precursor as the HIL under identical conditions.

### 2.4. Characterization

The crystal phases of the NiO/ZnS nanostructures were analyzed using an X-ray diffractometer (MiniFlex2, Rigaku, Tokyo, Japan). High-resolution transmission electron microscopy (TEM) was used to measure the particle sizes of NiO/ZnS nanostructures and QDs (JEM-2100F, JEOL, Tokyo, Japan). X-ray photoelectron spectroscopy (XPS; Nexsa, Thermo Fisher Scientific, Waltham, MA, USA) was used to analyze the chemical composition of NiO/ZnS. The photoluminescence (PL) spectra of the QDs on various HILs were collected by a PL spectrophotometer (PS-PLEU-X1420, PSI, Yongin, Republic of Korea). The EL spectra of the QLEDs were measured using a spectroradiometer (CS 2000, Minolta, Osaka, Japan), while the current density–voltage–luminance (J–V–L) characteristics were obtained simultaneously by connecting the spectroradiometer to a source meter (Keithley 2400, Keithley, Cleveland, OH, USA). All measurements were carried out under ambient conditions.

## 3. Results and Discussion

**Figure 2:**
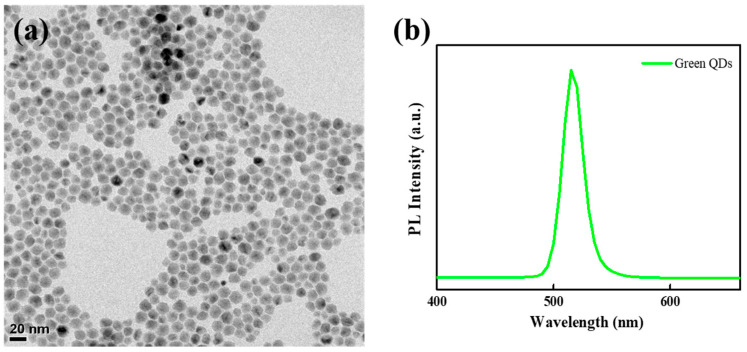
(**a**) TEM image and (**b**) PL spectrum of QDs.

The CdZnSeS as a core was first synthesized through a hot injection method and were further used to produce CdZnSeS/ZnS QDs by consecutively over-coating with additional ZnS shell. As a result, this CdZnSeS/ZnS QDs with a core/shell structure dramatically improve the photoluminescence quantum yield [20]. Figure 2 shows highly efficient green-emitting CdZnSeS/ZnS QDs, with an average size, emission peak wavelength, and full-width-at-half-maximum of 12.0, 515, and 19.4 nm, respectively.

**Figure 3:**
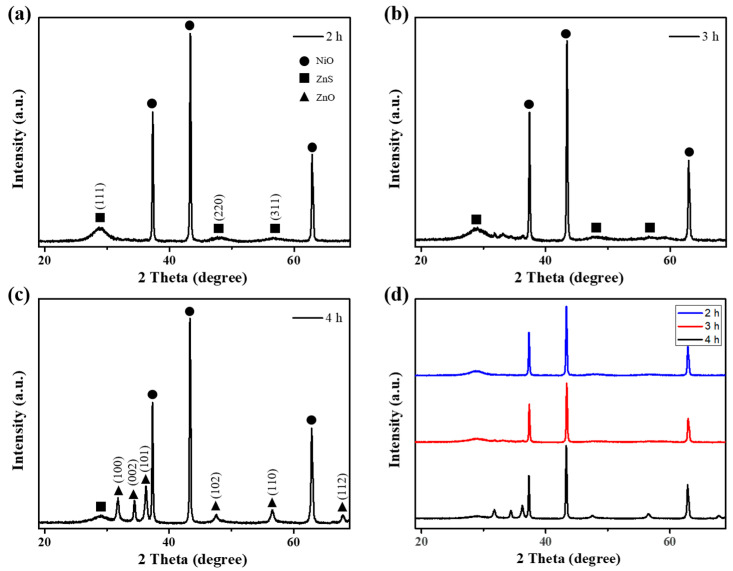
XRD patterns of NiO/ZnS nanostructures after a reaction time of (**a**) 2h, (**b**) 3h, and (**c**) 4 h. (**d**) Comparison for all peaks position.

Figure 3 shows the X-ray diffraction (XRD) patterns of the NiO/ZnS nanostructures after different reaction times. When the synthesis was performed in the presence of Na_2_S and ZnCl_2_, all the diffraction peaks of the ZnS shell were well indexed to the cubic zinc blende structure (ICSD #98-005-2223). The XRD pattern (Figure 3a) presents peaks at 2θ = 28.70°, 47.75°, and 56.67° corresponding to the (111), (220), and (311) crystal planes of ZnS, respectively. As the reaction time increased, the intensity of the ZnS diffraction peaks decreased significantly and new peaks corresponding hexagonal wurtzite zinc oxide (ZnO) (ICSD #98-005-7478) appeared in the NiO/ZnS nanostructure. This indicates that ZnO covers the shallow surface of the ZnS shell as reaction time increases. After a reaction time of 4 h, ZnO was the dominant component in the shell. The reduced ZnS peak also indicates that ZnO was formed on the outside of the shell. Figure S1 shows a TEM image of NiO/ZnS nanostructures, from which their average diameter was determined to be approximately 9.5 nm.

**Figure 4:**
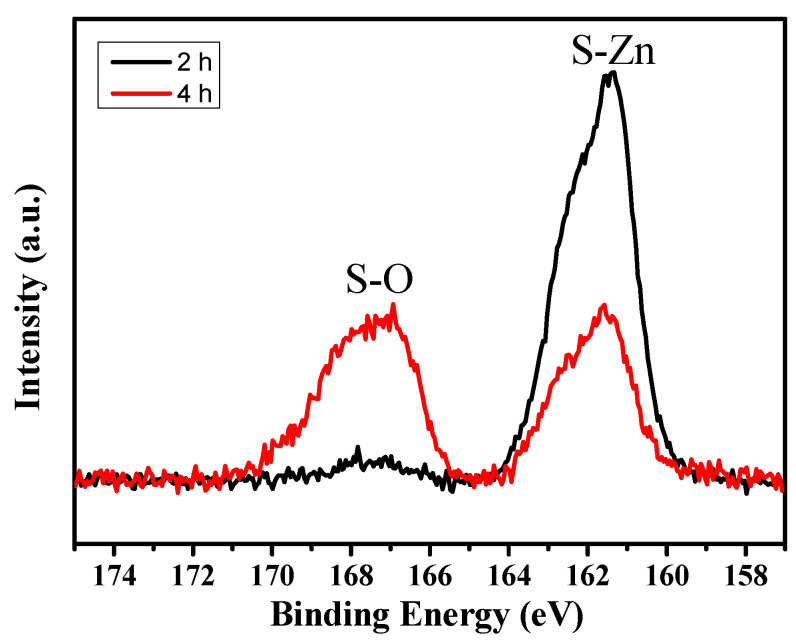
S 2p XPS spectra of NiO/ZnS nanostructures at different reaction times.

To further investigate the chemical state of the NiO/ZnS nanostructure according to the reaction time, the S 2p XPS spectra of NiO/ZnS are shown in Figure 4. The spectra contain two isolated bands centered at 167.4 and 161.6 eV, which can be attributed to the S–O and S–Zn bonds, respectively [21]. The intensity of the XPS peak corresponding to S–Zn decreased with increasing reaction time, whereas that corresponding to S–O increased. The transformation of the initial ZnS layer into ZnO is attributed to the adsorption of hydroxyl species on the surface, which induces changes in the chemical composition and structure of ZnS over time [22]. As a result of the decomposition of the ZnS layer with increasing reaction time, sulfoxides (SO_x_), in addition to ZnO, were formed, as confirmed by the XPS analysis.

**Figure 5:**
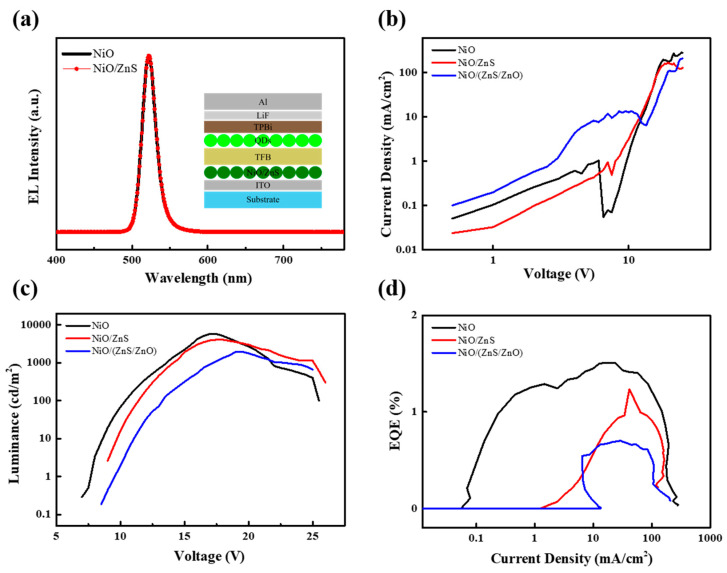
(**a**) EL spectra, (**b**) current density–voltage, (**c**) luminance–voltage, and (**d**) EQE–current density of QLEDs with NiO, NiO/ZnS, or NiO/(ZnS/ZnO) as the HIL. Inset shows the device structure of QLEDs.

Figure 5 presents the optoelectronic characteristics of the QLEDs with NiO/ZnS (reaction time: 2 h) and NiO/(ZnS/ZnO) (reaction time: 4 h) as the HIL. Devices with NiO without the ZnS shells were also fabricated to compare their properties. The QLEDs consisted of patterned ITO/HIL (NiO/ZnS, NiO/(ZnS/ZnO) or NiO)/TFB/QDs/TPBi/Al (Inset of Figure 4a). The QLED structure was designed to achieve an efficient carrier injection and charge balance in the QD EML. Generally, holes are difficult to transport to QD EML because of the large injection barrier between the VBM of the inorganic HTL and the QD layer. Since the highest occupied molecular orbital level of TFB is located between the VBM of NiO/ZnS and QDs, it is utilized as the HTL to reduce the energy barrier between them in this standard structure. Because TFB thin films are chemically and physically stable in nonpolar solvents, QDs can be simply spin-coated on top of the TFB layer. As shown in Figure 5a, no parasitic peaks from the two neighboring layers (TFB and TPBi) were observed in the EL spectra of either device, indicating that he presence of the ZnS shell does not affect the charge balance in the EML. Interestingly, Figure 5b shows that the devices with NiO/ZnS exhibited a lower leakage current in the ohmic region and a similar current density above the turn-on voltage. Observing Figure 5b,c shows that the thin ZnS shell limits the current flow in the low-voltage region but does not interfere with the light-emitting performance of the QLEDs in the operational voltage region. Therefore, similar results from peak luminance and EQE (5808.6 cd/m^2^, and 1.51% for QLEDs with NiO and 4068.5 cd/m^2^, and 1.23% cd/A for QLEDs with NiO/ZnS, respectively) were obtained. QLEDs with NiO/(ZnS/ZnO) exhibited poor performance with a high leakage current in the ohmic region, which can disturb the balanced charge transport to EML in the low voltage region.

**Figure 6:**
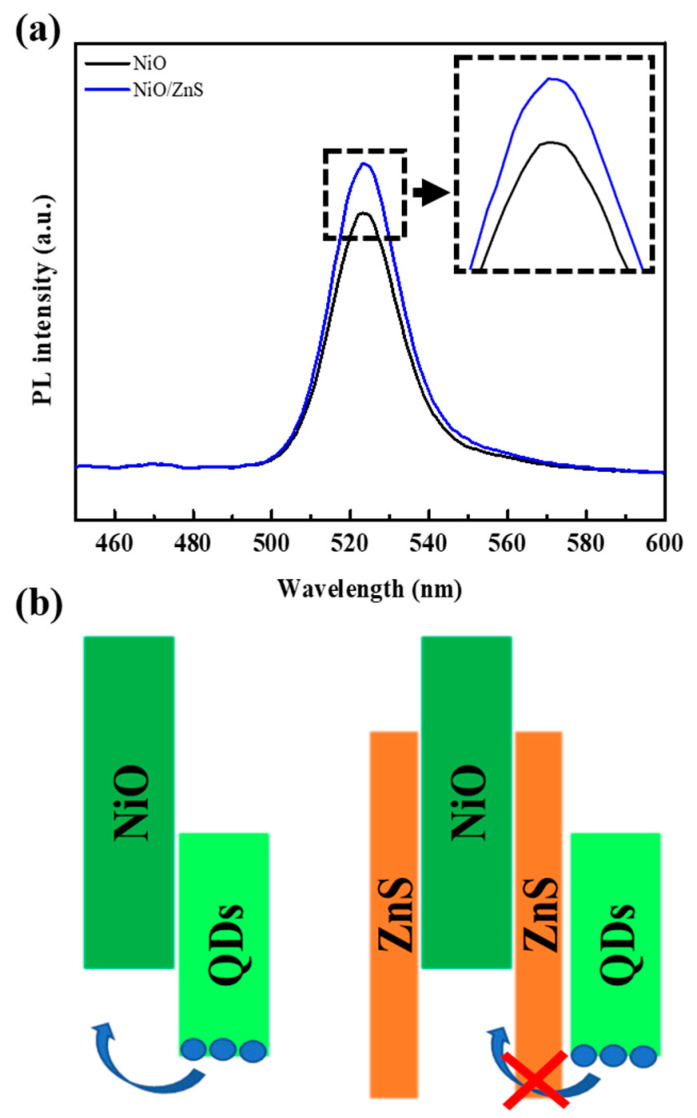
(**a**) PL spectra of glass/(NiO/ZnS)/QDs and glass/NiO/QDs. (**b**) Illustrated scheme for exciton quenching induced by the charge transfer process.

Figure 6 shows the steady PL spectra of the two samples with glass/NiO/QDs and glass/(NiO/ZnS)/QDs structures. For the future all-inorganic devices, the exciton quenching phenomenon at interfaces (inorganic materials/QDs) was investigated. The PL intensity of the QDs decreased significantly when they were in contact with the bare NiO. This is because the excitons are easily quenched by the interfacial charge transfer process at the NiO/QD interface [23]. However, with the core–shell structure of NiO/ZnS, the PL intensity was greatly enhanced, indicating that the quenching processes can be effectively suppressed with the ZnS shell. This is because the lower VBM level of ZnS can effectively block the interfacial charge (mainly holes) transfer, as shown in Figure 6b. In addition, the ZnS shell of NiO/ZnS was the same as that of the QDs. When two adjacent materials have the same composition and crystal structure, the number of non-radiative recombination sites to quench excitons can be reduced at the interface [24]. Figure S2 presents the PL spectra of glass/NiO/TFB/QDs and glass/(NiO/ZnS)/TFB/QDs structures. Because of the strong PL peak from TFB below 450 nm, it was difficult to attribute the small difference between the two PL peaks to the ZnS shell.

The performance of QLEDs with NiO-based HIL is not compatible with devices using PEDOT:PSS. Therefore, the high temperature annealing (over 400 °C) was applied to improve the charge transport in HIL [14,18,25]. Since that annealing can damage the ITO electrode or other layers in the device, the deposition process of NiO/ZnS in this work was performed below 100 °C despite their relative low performance.

## 4. Conclusions

QDs possess unique properties that render them ideal candidates for optoelectronic devices. QLEDs utilizing QDs as the emission layer require simplified fabrication processes compared with vacuum-based systems. However, the practical application of PEDOT:PSS as a HIL is limited because of its acidic and hygroscopic nature. This study explored NiO/ZnS with efficient hole injection and electron blocking capabilities. The synthesis process for adding the ZnS shell to NiO was investigated based on the reaction time in a sophisticated manner. The resulting NiO/ZnS HIL demonstrated promising potential for use in all-inorganic QLEDs, exhibiting a peak luminance and EQE of 4068.5 cd/m² and 1.23%, respectively. Furthermore, the presence of the ZnS shell suppressed exciton quenching, leading to ameliorated PL intensity. Overall, NiO/ZnS has emerged as a viable inorganic material for HIL in QLEDs, facilitating future advancements in display technology.

## Data Availability

The data presented in this study are available on request from the corresponding author.

## Supplementary Materials

The following supporting information can be downloaded at: https://www.mdpi.com/article/10.3390/ma16145106/s1, Figure S1: A TEM image of NiO/ZnS nanostructures; Figure S2: PL spectra of glass/(NiO/ZnS)/TFB/QDs and glass/NiO/TFB/QDs.
