# Supplementary material for: Structural and Optical Properties of NiO/ZnS Core–Shell Nanostructures for Efficient Quantum Dot Light-Emitting Diodes
Source: Materials (Basel). 2023 Jul 20;16(14):5106. doi: 10.3390/ma16145106 (PMC10383065; doi:10.3390/ma16145106)
Supplement: Supplementary file 1 [file materials-16-05106-s001.zip › materials-2467526-supplementary.pdf]

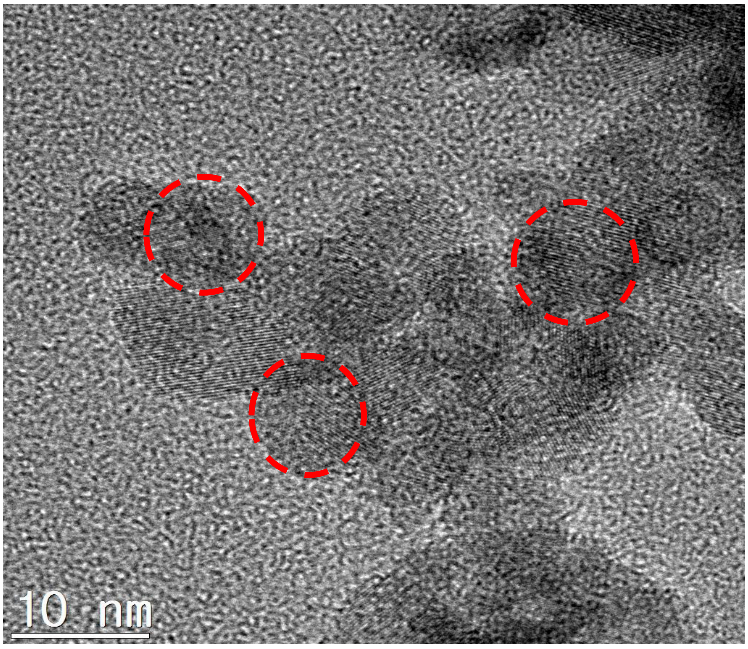

Figure S1. A TEM image (scale bar, 10 nm) of NiO/ZnS nanostructures.

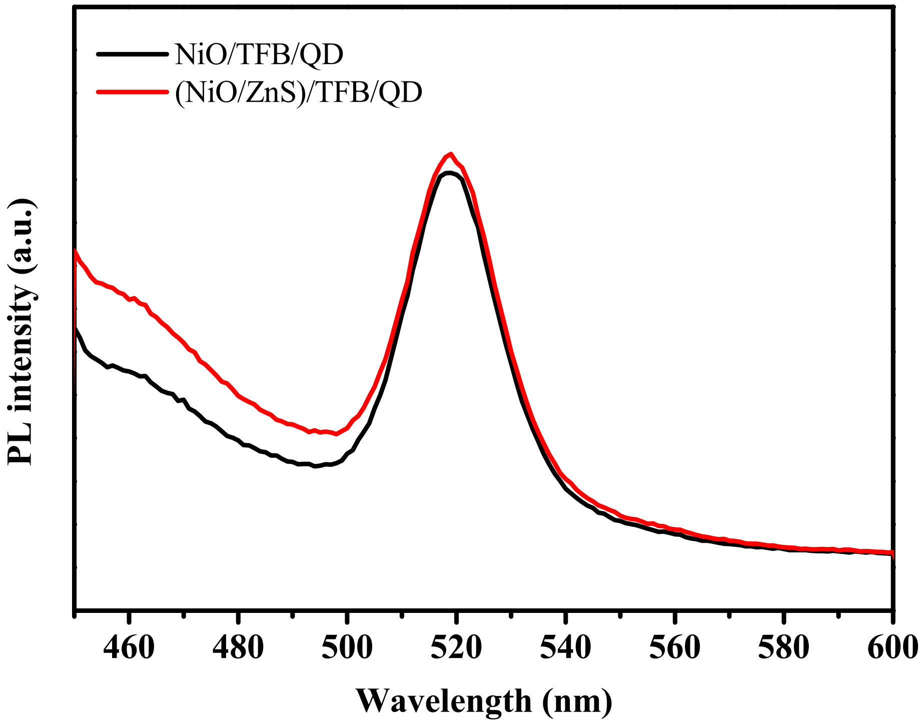

Figure S2. PL spectra of glass/(NiO/ZnS)/TFB/QD and glass/NiO/TFB/QD.
